# Supplementary material for: Tree Species and Epiphyte Taxa Determine the “Metabolomic niche” of Canopy Suspended Soils in a Species-Rich Lowland Tropical Rainforest
Source: Metabolites. 2021 Oct 21;11(11):718. doi: 10.3390/metabo11110718 (PMC8621298; doi:10.3390/metabo11110718)
Supplement: Supplementary file 1 [file metabolites-11-00718-s001.zip › metabolites-1339386-supplementary.pdf]

SUPPLEMENTARY MATERIAL

Table S1. Processing parameters of LC-MS chromatograms using MzMine 2.0 (Pluskal et al., 2010). Chromatogram represents by the total ion current (TIC).

		(+H) Chromatograms		(-H) Chromatograms
1	**Baseline correction**			
	Chromatogram type	TIC		TIC
	MS level	1		1
	Smoothing	10E6		10E6
	Asymmetry	0.001		0.001
2	**Mass detection** (Exact Mass)			
	Noise level	4.5×10^5		4.5×10^5
3	**Chromatogram builder**			
	Min time span	0.05		0.05
	Min height	25000		25000
	m/z tolerance	0.002		0.002
4	**Smoothing**			
	Filter width	5		5
5	**Chromatogram deconvolution (Local minimum search)**			
	Chromatographic threshold	70%		70%
	Search minimum in RT range (min)	0.1		0.1
	Minimum relative height	7.0%		7.0%
	Minimum absolute height	30000		30000
	Min ratio of peak top/edge	2		2
	Peak duration range	0.0-2.0		0.0-2.0
6	**Chromatogram alignment (join alignment)**			
	m/z tolerance	0.001		0.001
	weight for m/z	80		80
	RT tolerance	0.3		0.3
	Weight for RT	20		20
7	**Gap filling (Peak Finder)**			
	Intensity tolerance	20%		20%
	m/z tolerance	0.001		0.001
	Retention time tolerance	0.1		0.1
	RT correction	marked		marked
8	**Filtering**			
	Minimum peaks in a row	25		25
	**Ions excluded from database**	<75<br>Between 0.0 and 1 min<br>Between 28.5 and 30 min		<85<br>Between 0.0 and 1,1 min<br>Between 27.0 and 30 min

“

Pathway enrichment analysis
floor soil versus
suspended soil without epiphytes

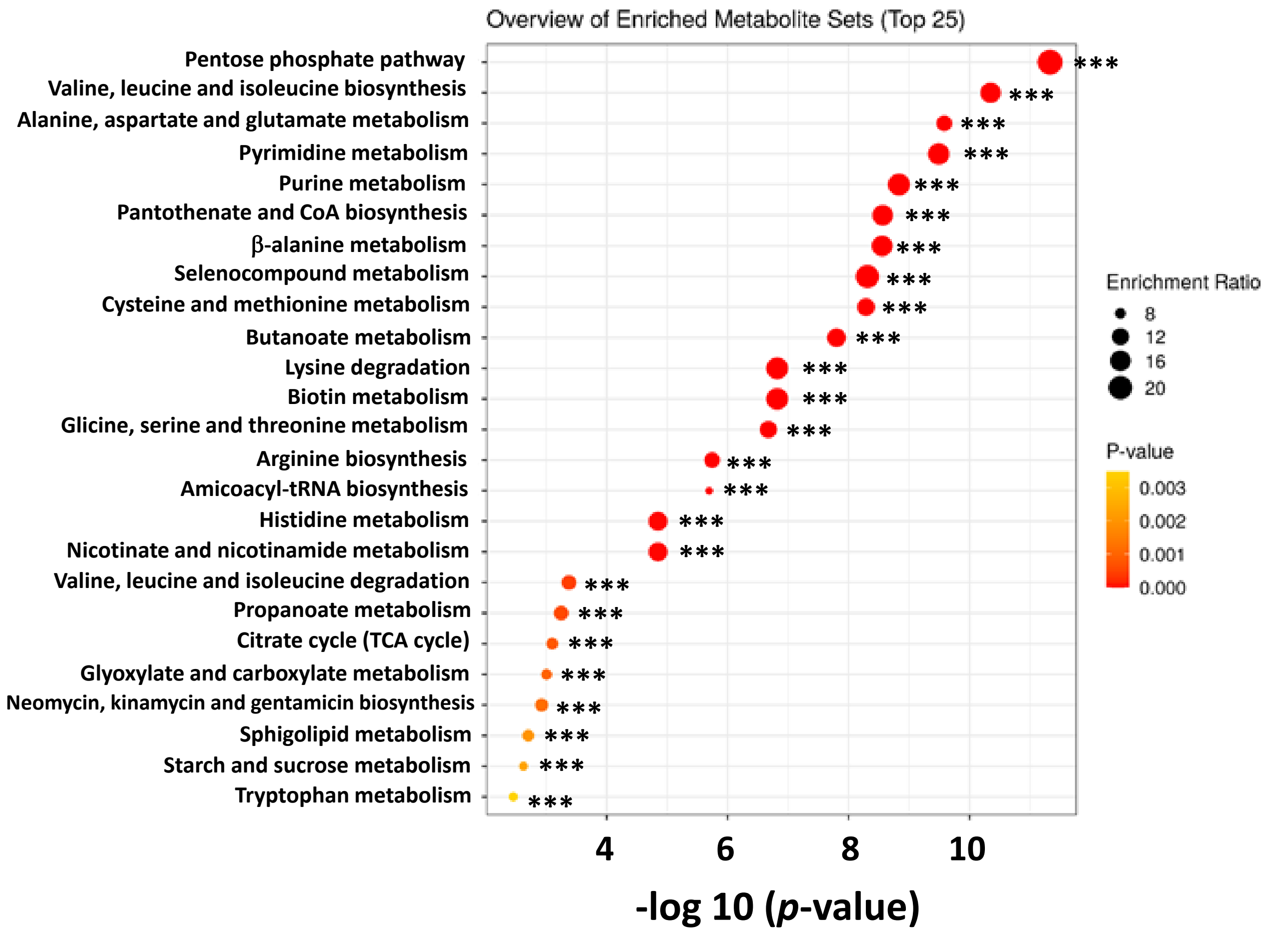

Figure S1. Enrichment pathway analysis of soil metabolomics profile versus suspended soils without epiphytes. *** ($P<0.001$).
